# Supplementary material for: Functions of Enyolreductase (ER) Domains of PKS Cluster in Lipid Synthesis and Enhancement of PUFAs Accumulation in Schizochytrium limacinum SR21 Using Triclosan as a Regulator of ER
Source: Microorganisms. 2020 Feb 21;8(2):300. doi: 10.3390/microorganisms8020300 (PMC7074904; doi:10.3390/microorganisms8020300)
Supplement: Supplementary file 1 [file microorganisms-08-00300-s001.pdf]

Supplementary Material

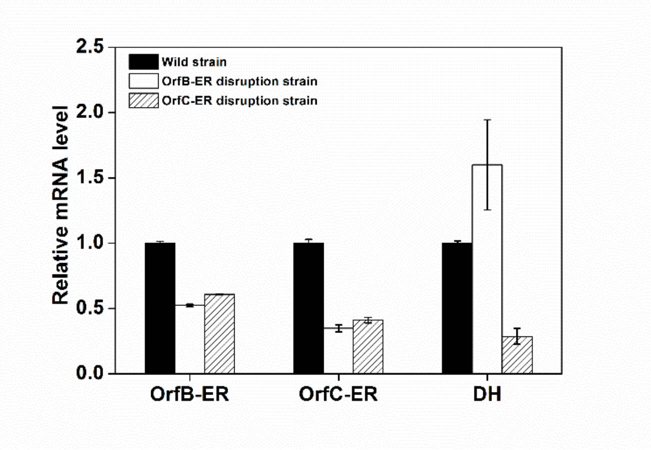

Figure S1. The gene expression profile of the OrfB-ER, OrfC-ER and DH at maximum lipid content period by RT-PCR from wild strain, OrfB-ER gene disruption strain and OrfC-ER gene disruption strain. OrfB-ER means ER gene located on OrfB gene cluster; OrfC-ER means ER gene located on OrfC gene cluster; DH means beta-hydroxyacyl-acyl carrier protein dehydratases.

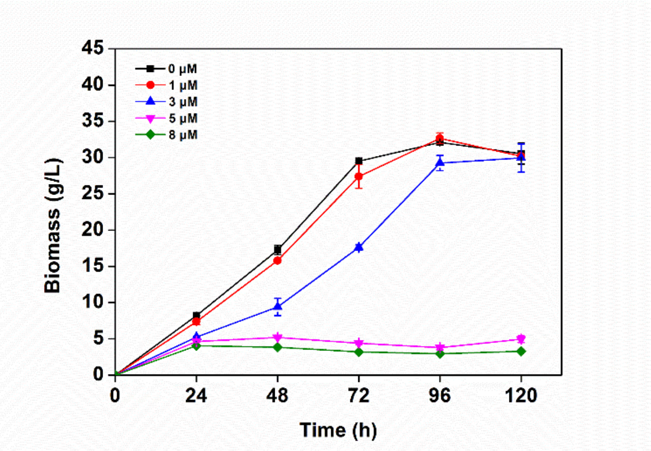

(a)

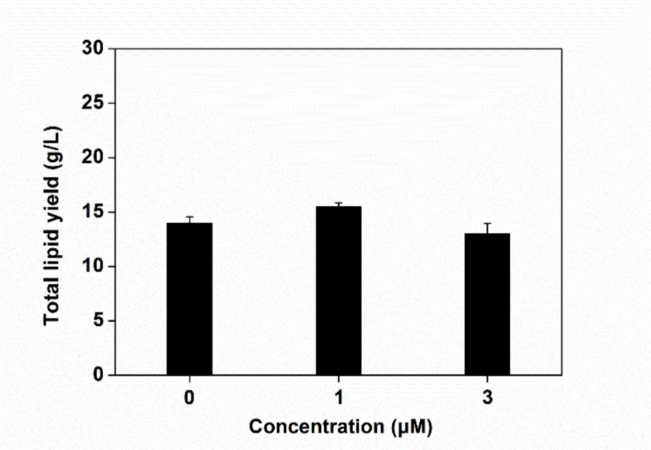

(b)

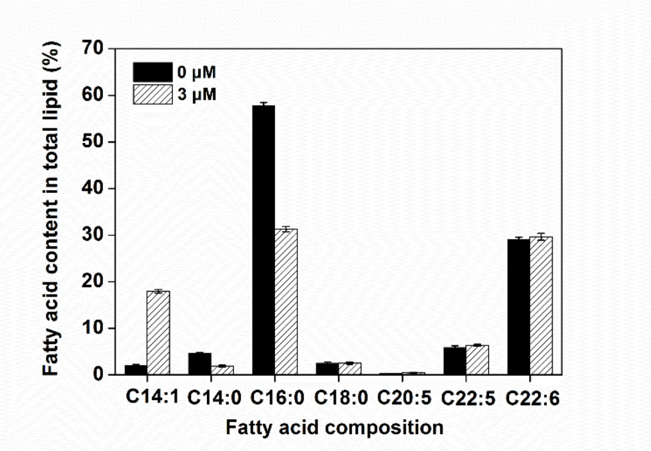

(c)

Figure S2 Effect of different concentration of triclosan added at 0 h on (a) Biomass, (b) Total lipid yield at 120 h, and (c) Fatty acid content in total lipid at 72 h.

Table S1. Primers used in vector construction and PCR verification of mutants

Primers	Sequence(5′ -3′)
B-UF (A)	AGGGGTACCGGATCTTGGCAGCCGTGCCTTCATG
B-UR (B)	CGCATCGATCGAGCTGCGGGGTCAAGGTCATAAA
B-DF (C)	CGTCTGCAGTGGCCCCAGCAGCTGACATG
B-DR (D)	GCCGGATCCCGCAAGAAGCAGGCACCACG
C-UF (E)	AAGTCGACATGCTGACCTTGTGATTGC
C-UR (F)	AAGTCGACATGCTGACCTTGTGATTGC
C-DF (G)	GTCCTGCAGTCTCATCAACCTCCGCAACC
C-DR (H)	CGCGGGATCCAGGCATGGACTCGAAGGA
ZEO-F	CTTCAAAACACCCAAGCACAGCA
ZEO-R	GTGGACACGACCTCCGACCACTC
B-ER-F	AAGGATCTTGGCAGCCGTGC
B-ER-R	GAGGCGGCGCAAGAAGCA
C-ER-F	CTCGGTGATGAAGGCTTCATG
C-ER-R	GGACGGGAACATGGTTCCC

Table S2. OrfB sequence of PKS gene cluster in *Schizochytrium limacinum* SR21

GATCGATCTAAAAGTGCCGGTCAGGTGACACGCAAGCTCTTTTTTTGTTTACAGTAA
GCAGCAACAAGAAAGCAAAAAGATGGCCTCTCGCAAGAATGTGAGCGCTGCTCAC
GAAATGCACGACGAGAAGCGCATTGCCGTGGTGGGCATGGCCGTGCAATACGCGG
GCTGCAAAGACAAGGAAGAGTTCTGGAAAGTAGTCATGGGCGGTGAGGCTGCATG
GACTAAGATTAGCGATAAACGCCTCGGATCCAACAAGCGAGCCGAGCACTTCAAA
GCAGAGCGTAGCAAATTTGCAGATACCTTTTGCAACGAGAACTACGGCTGCGTCGA
TGACTCCGTCGATAACGAACACGAGCTTCTCCTTAAGCTCTCCAAGAAGGCTCTCTC
CGAGACATCGGTCTCCGACTCTACAAGGTGCGGTATTGTGAGCGGATGCCTGTCCTT
TCCCATGGACAACCTCCAGGGCGAACTCCTCAATGTGTACCAAAACCACGTCGAA
AAGAAACTCGGCGCTCGCGTCTTCAAGGATGCCTCCAAGTGGTCCGAGCGTGAGC
AGTCGCAGAACCCCGAGGCTGGTGACCGCCGCATCTTTATGGACCCGGCATCCTTC
GTAGCAGAAGAGCTCAACCTCGGTCCTCTTCACTACTCTGTCGATGCTGCCTGTGCC
ACCGCCCTTTACGTCCTTCGCCTCGCCCAGGACCACCTCGTTTCCGGTGCTGCTGAT
GTCATGCTCGCTGGTGCAACTTGCTTCCCGGAGCCCTTTTTCATTCTCTCCGGATTCT
CCACTTTCCAGGCCATGCCTGTATCGGGAGACGGCATCTCGTACCCGCTTCACAAG
GACAGTCAGGGTCTCACCCCTGGTGAAGGTGGTGCCATTATGGTTCTCAAGCGCCTT
GACGACGCTATTCGCGATGGAGACCACATTTACGGTACTCTGCTCGGTGCTACCATC
AGCAATGCTGGCTGTGGTCTTCCCCTCAAGCCGCACTTGCCCAGCGAGAAGTCCTG
CCTCATTGATACCTACAAGCGCGTCAACGTGCACCCGCACAAGATCCAGTACGTCG
AGTGCCACGCAACGGGTACTCCCCAGGGAGACCGCGTTGAGATTGATGCCGTCAA
GGCTTGCTTCGAGGGCAAGGTGCCTCGCTTTGGAAGCTCCAAGGGTAACTTTGGCC
ACACACTCGTTGCAGCTGGTTTCGCAGGCATGTGCAAGGTACTCCTTGCCATGAAG
CATGGTGTGATCCCGCCCACTCCTGGTGTCGATGGATCTTCCCAAATGGACCCGCTT
GTGGTCTCTGAGCCCATCCCATGGCCCGACACTGAGGGCGAGCCCAAGCGCGCTG

GTCTCTCCGCTTTCGGCTTTGGTGGCACCAACGCCCACGCAGTCTTTGAGGAGTTTG
ACCGCTCCAAGGCTGCCTGTGCCACCCACGATAGCATCAGTTCCCTCAGCTCACGTT
GTGGCGGGGAGGGCAACATGCGCATTGCTATTACCGGTATGGATGCCACCTTCGGCT
CCCTCAAGGGCCTGGACGCCTTTGAGCGTGCCATCTACAATGGCCAACATGGTGCT
GTGCCATTGCCTGAGAAGCGCTGGCGTTTCCTTGGTAAAGACAAGGACTTTTTGGA
CCTGTGCGGTGTCAAGGAGGTGCCCCACGGATGCTACATTGAGGACGTCGAGGTGG
ACTTTAGCCGCCTGCGCACGCCCATGACGCCAGACGACATGTTGCGCCCCATGCAG
CTACTTGCTGTCACAACCATCGACCGTGCCATTCTCAACTCTGGCCTCAAGAAGGG
AGGTAAGGTCGCTGTCTTCGTCGGCCTTGGCACTGACCTTGAGCTCTACCGTCACCG
CGCCCGCGTTGCCCTCAAGGAGCGTGCTCGTCCCGAAGCCGCTTCAGCCCTCAATG
ATATGATGTCCTACATCAACGATTGCGGTACCGCTACCTCGTACACATCCTACATCGG
CAACCTCGTGGCCACCCGCGTGTCTTCACAATGGGGTTTCGAGGGTCCTTCTTTCAC
CATCACAGAGGGCAACAACTCCGTCTACCGTTGCGCAGAGTTGGGCAAGTACTTGC
TCGAGACTGGCGAGGTCGAGGCCGTAGTGATCGCCGGTGTGGATCTTTGCGCCAGC
GCTGAGAATCTCTACGTGAAGTCGCGTCGTTTCAAGGTCTCGGAGCAGGAGAGCCC
GCGGGCCAGCTTCGACTCCGGCGCTGACGGCTACTTTGTTGGTGAGGGATGTGGTG
CCCTCGTCCTCAAGCGCGAGAGCGACTGCACCAAGGACGAACGCATTTACGCCTG
CATGGACGCTATCGTGCCCGGCAACATGCCGGCAGCCTGCATGGAGGAGGCTCTCG
CCCAGGCTCGCGTCAACCCCAAGGACGTTGAGATGCTCGAGCTCTCCGCTGACTCT
GCCCGCCACCTCAAGAACCCCTCCGTTCTGCCTAAGGAACTCACTGCTGAGGAGGA
AATCCGCGGCATTGAGGCCATTCTCAGCCAGCGCTCTAGCAACGAAGCTGTGGAGC
CCCACAACGTCGCTGTCAGCAGCGTCAAGTCCACTGTCGGTGACACCGGCTACGCC
TCAGGAGCTGCCAGTCTCATCAAGACGGCTCTCTGTCTGTACAACCGCTACTTGCCC
TCAAACGGCGCCTCCTGGGAGGAGCCTGCACCTGAGACACAGTGGGGCAAGTCTC
TGTACGCGTGCCAGTCCTCGCGGGCCTGGTTGAAGAACCCTGGAGCTCGCCGCCAC
GCAGCTGTCTCAGGTGTTTCCGAGACCCGTTCATGCTACACGGTGCTGCTCTCTGAT
GTGGAGGGCCACCACGAGACCAAGAGCCGCATTTCGCTCGATGACGATGCCGTCA
AACTCCTCGTAATCCGCGGAGACTCCCATGACGCTATCACGCAGCGTGTTGACAAG
CTCCGCGAGCGCCTCGCCCAGCCTAGCGCTAATGTACGTCTTGCTTTTATGGAGTTG
CTCGGCGAGAGCATTGCCCAGGAGACCAAGACCCCGTTGCCGGCCTTCGCTCTGTG
CCTGGTGACCTCTCCTAGTAAGCTCCAGAAGGAGCTTGAACTCGCCTCCAAGGGCA
TCCCGCGGAGTCTTAAGATGGGCCGCGACTGGACATCACCCTCGGGCAGCCACTTT
GCACCCAAGCCACTGTCAAGCGATCGCGTTGCGTTTATGTACGGCGAAGGCCGAAG
CCCTTACTATGGTATCGGCCTTGACATTCACCGCATCTGGCCCGAACTTCACGAGTTT
GTAAACGCCAAGACCAACAAGCTTTGGGATCAAGGCGACAGATGGTTGATCCCGC
GCGCCTCGACGAAGGAGGAGCTTAAGGCGCAGGAAGATGAGTTCAACCGCAACC
AGGTGGAGATGTTCCGACTCGGTATTCTCATGTCCATGTGCTTCACCCACATCGCTC
GCGACGTGCTTGGCATCCAGCCCAAGGCTGCTTTCGGACTGAGCCTTGGAGAGATT
TCCATGGTTTTTGCCTTTTCTGAGAAGAACGGCCTTGTCTCTGAGGAGCTGACAACT
AAACTCCGCAACTCGGAGGTCTGGCGTAAGGCCCTCGCTGTTGAGTTTGACGCCCT
CCGCAAGGCCTGGAATATTCCCCAAGATACCCCTGTCAGCGAGTTCTGGCAAGGAT
ACGTGGTACGTGGAACCCGCGAGGCCGTTGAAGCGGCCATCGGCCCCAACAATAA
GTACGTGCACTTGACCATTGTCAACGATGCCAACAGTGCTCTCATCAGTGGCAAGC
CTGAAGATTGCAAGGCTGCCATTGCTCGCCTGAGCAGCAACCTCCCTGCTTTGCCC

GTGGACCTTGGTATGTGTGGCCACTGCCCCGTGGTCGAGCCGTACGGCAAGCAGAT
CGCTGAGATCCATAGCGTCCTCGAGATTCCCGAGGTTGCCGGCCTTGACCTGTACAC
GAGCGTCAACCAGAAGAAGCTTGTTAACAAGTCCACTGGAGCCAGCGACGAGTAC
GCACCCAGCTTTGGTGAATACGCAGCACAGCTGTACACTGTTCAGGCAGACTTTCC
TAAGATCGCCAAGACCGTTAGCGACAAGAACTTTGACGTCTTTGTTGAGACTGGTC
CCAACGCCCACCGTAGCGCCGCAATTCGCGCCACCCTTGGAAATAGCAAGCCTTTT
GTCACCGGATCCATGGACCGCCAGAACGAGAATGCTTGGACAACCATGGTCAAGC
TGGTTGCCTCTCTCCAAGCCCACCGCGTGCCTGGCGTGAAGGTCTCCCCTCTGTACC
ACCCCGAGACTGTTGAGGAGGCTACGCAGAGTTACAACGATATGGTGGCTGGCAA
GAAGCCTACTAAGAACAAGTTCTTGCGTAAGATTGTGGTCAATGGTCGCTATGACCC
CAAAAAGCAGCTCGTGCCGCCCCAGGTGCTAGCTAAGCTTCCTCCTGCGGACCCCA
AGATCGAGGCTCTTATCCAGGCTCGCAAGATGCAGCCTATTGCCCCCAAGTTCATGG
AGCGTCTCGACATTCAGGAGCAAGACGCCACACGCGACCCTATTCTCAACAAGGAT
AACAAACCTTCCGCTGCTCCTGCCCTTGCCCCTGCTGCTCCGGCCCGCAGCGTCTCC
GGAGCTGTTGTGGCTTCCTCTGAGGCTCTCCGTGCCAAACTTTTGGAGCTCAACAGC
ACTTTGATGCTTGGTGTCAACGCCAACGGTGATCTCGTTGAAGCAAGCCCAAGTGA
AGCATCTATTGTTGTGCCCAAGTGCGATATCAAGGATCTTGGCAGCCGTGCCTTCAT
GGAGACATATGGTGTATCCGCCCCCATGTACACCGGCGCCATGGCAAAGGGCATTG
CATCCGCTGAGATGGTTATCGCTGCCGGAAAGCGCGGCATCCTTGGTTCTCTCGGTG
CTGGTGGTCTTCCTATCGCCACCGTACGCAAGGCTCTCGAAGCTATCCAGGCTGAAC
TGCCCAAGGGCCCTTACGCTGTCAACCTCATCCACTCTCCCTTCGACAGCAACCTCG
AGAAGGGTAACGTCGACCTCTTCCTCGAGAAGGGCGTCACTGTCGTTGAAGCCTCC
GCCTTTATGACCTTGACCCCGCAGCTCGTGCGCTACCGTGCTGCAGGTCTCTCTCGC
GCTGCTGATGGCTCCACGGTTATTAAGAACCGCGTCATCGGTAAGGTTTCTCGCACA
GAGCTTGCCGCAATGTTTATCCGTCCCGCGCCCGAGAATCTCCTCGAGAAGCTGCTG
AAGTCCGGCGAGATCACCCAAGAGCAGGCTGCTCTCGCACGCACAGTGCCTGTGG
CAGACGACATTGCCGTTGAGGCGGACTCCGGTGGCCACACCGATAACCGCCCCATC
CACGTCATCCTCCCTCTCATTGTCAACCTCCGTGATCGTCTGCACAAGGAGTGCGGC
TACCCTGCCCACCTTCGCGTTCGCGTTGGTGCTGGTGGTGGCATTGGATGCCCTCAG
GCCGCCATTGCCACCTTCAACATGGGCGCGGCCTTCATCGTCACTGGTACCGTAAAC
CAGATGAGTAAGCAAGCTGGAACCTGTGACACCGTTCGCAAGCAGCTCTCACAAG
CCACCTACTCCGACATCTGCATGGCCCCAGCAGCTGACATGTTTGAGGAAGGTGTC
AAGCTCCAGGTGCTCAAGAAGGGAACTATGTTCCCCTCGCGTGCCAACAAGCTCTA
TGAGCTCTTCGTCAAGTATGACTCCTTTGAGTCCATGGCTCCTGGAGAGCTGGAACG
TGTGGAGAAGCGCATTTTCAAGAAGTCTCTGTCAGAAGTTTGGGAAGAGACCAAG
GACTTCTACATCAACAGGTTGCAGAACCCGGAGAAGATTGAGCGCGCGGAGCGTG
ACCCCAAGCTTAAGATGTCCTTGTGCTTCCGCTGGTACCTTGGTTTGGCGAGCTTCT
GGGCAAACGCTGGCATCCCGGACCGTGCCATGGACTACCAGGTTTGGTGTGGCCCA
GCGATTGGATCTTTCAACGACTTCATCAAGGGTACCTACCTTGACCCCGCCGTTGCC
AACGAGTACCCCGATGTTGTGCAAATCAACTTGCAGATCCTCCGTGGTGCCTGCTTC
TTGCGCCGCCTCGAAGCTGTCCGTAATGCCCCGCTGAAGGCTAACGCCAAGCAGGT
TGCTGCCGAGATTGATGACATCTACGTGCCCACTGAGCGCCTGTAAGCCCAATTTGC
TCCTGATCTGTTCCCATGATTATGATAGGGATAGGTAGTAGTTATAGCTAGACTCATT
CCATTCACTTAATCCACATATGCAAATTATAATTTTATGTGTCGC

Table S3. OrfC sequence of PKS gene cluster in *Schizochytrium limacinum* SR21

ATGACCAAGGAGGAGCTCACCAGCGGCAAGAACGTCGTTTTCGACTATGACGAGC
TCCTTGAGTTCGCCGAGGGTGACATCAGCAAGGTCTTCGGCCCCGAATTCAGCCAG
ATCGACCAGTACAAGCGTCGCGTTCGTCTCCCCGCCCGCGAGTACCTCCTCGTCAC
CCGCGTCACCCTCATGGACGCCGAGGTCAACAACTACCGCGTCGGTGCCCGCATGG
TCACTGAGTACGACCTCCCCGTCAACGGTGAGCTCTCTGAGGGTGGTGACTGCCCC
TGGGCCGTGCTCGTCGAGAGTGGTCAGTGTGATCTCATGCTCATCTCCTACATGGGT
ATTGACTTCCAGAACAAGAGCGACCGCGTCTACCGTCTGCTCAACACCACCCTCAC
CTTCTACGGTGTTGCCCAGGAGGGCGAGACCCTGGAGTACGACATCCGCGTGACCG
GCTTCGCCAAGCGTCTCGACGGTGACATCTCCATGTTCTTCTTCGAGTACGACTGCT
ACGTCAACGGCCGTCTCCTCATCGAGATGCGCGACGGCTGTGCCGGTTTCTTCACC
AACGAGGAGCTCGCCGCCGGCAAGGGTGTCGTCTTTACCCGCGCTGATCTCCTCGC
CCGCGAGAAGACCAAGAAGCAGGACATCACCCCGTACGCCATTGCCCCGCGTCTT
AACAAGACCGTTCTCAACGAGACTGAGATGCAGTCCCTCGTGGACAAGAACTGGA
CCAAGGTTTTCGGCCCCGAGAACGGCATGGACCAGATCAACTACAAACTCTGCGCC
CGTAAGATGCTCATGATTGACCGCGTCACCAAGATTGACTACACCGGTGGCCCCTA
CGGCCTTGGTCTTCTCGTTGGTGAGAAGATCCTCGAGCGCGACCACTGGTACTTTCC
GTGCCACTTCGTCGGAGACCAGGTCATGGCTGGATCCCTCGTGTCTGACGGCTGCA
GCCAGCTCCTCAAGATGTACATGCTCTGGCTCGGCCTCCACCTTAAGACCGGTCCCT
TCGACTTCCGCCCCGTCAACGGCCACCCCAACAAGGTCCGCTGCCGTGGCCAGATC
TCCCCGCACAAGGGTAAGCTCGTATACGTCATGGAGATCAAGGAGATGGGCTACGA
CGAGGCTGGTGACCCGTACGCCATCGCCGATGTCAACATTCTCGACATTGACTTCGA
GAAGGGCCAGACTTTCGACCTTGCCAACCTCCACGAGTACGGCAAGGGCGACCTC
AACAAGAAGATCGTCGTCGACTTCAAGGGTATTGCCCTCAAGCTCCAGAAGCGCTC
TGGCCCTGCCGTTGTCGCTCCCGAGAAGCCCCTCGCTCTCAACAAGGACCTTTGCG
CCCCGGCTGTTGAGGCCATCCCTGAGCACATCCTCAAGGGCGATGCTCTTGCCCCTA
ACCAGATGACCTGGCACCCGATGTCCAAGATCGCTGGCAACCCCACGCCCTCGTTC
TCTCCCTCGGCCTACCCTCCCCGTCCCATCACCTTCACCCCGTTCCCCGGCAACAAG
AACGACAACAACCACGTGCCCGGCGAGATGCCGCTCTCGTGGTACAACATGGCTG
AGTTCATGGCCGGCAAGGTCAGCCTCTGCCTCGGCCCTGAGTTCGCCAAGTTCGAT
GACTCCAACACCAGCCGCAGCCCTGCATGGGACCTTGCTCTTGTGACTCGTGTGGT
CTCCGTTTCTGACATGGAGTGGGTCCAGTGGAAGAACGTGGACTGCAACCCGTCCA
AGGGAACCATGGTTGGCGAGTTCGACTGCCCCATCGACGCCTGGTTCTTCCAGGGA
TCTTGTAACGACGGCCACATGCCGTACTCCATCCTCATGGAGATCGCCCTCCAGACC
TCTGGTGTCCTCACCTCTGTGCTCAAGGCCCCGCTCACCATGGAGAAGAAGGACAT
TCTCTTCCGCAACCTTGACGCCAACGCCGAGATGGTTCGCTCTGATATTGACCTCCG
CGGCAAGACCATCCACAACCTCACCAAGTGTACCGGCTACAGCATGCTCGGAGAC
ATGGGTGTCCACCGCTTCAGCTTCGAGCTCTCTGTTGATGGTGTAGTCTTCTACAAG
GGTACCACCTCCTTCGGCTGGTTCGTCCCTGAGGTCTTCATCTCCCAGACTGGTCTC
GACAACGGTCGCCGCACCCAGCCCTGGCACATTGAGTCCAAGGTGCCTTCCGCCCA
GGTCCTCACCTACGACGTTACCCCCAACGGTGCCGGTCGCACCCAGCTCTACGCCA
ACGCCCCCAAGGGCGCTCAGCTCACTCGCCGCTGGAACCAGTGCCAGTACCTTGAC

ACCATCGACCTTGTGGTCGCCGGTGGCTCCGCCGGTCTTGGCTACGGTCATGGCCGC
AAGCAGGTGAACCCCAAGGACTGGTTCTTCTCGTGCCACTTCTGGTTCGACTCCGT
CATGCCCGGCTCGCTCGGTGTGGAGTCTATGTTCCAGCTCGTCGAGTCCATCGCTGT
CAAGCAGGACCTCGCCGGCAAGTACGGCATCACCAACCCGACCTTCGCTCATGCTC
CGGGCAAGATCTCCTGGAAGTACCGTGGTCAGCTCACCCCCACCTCCAAGTTCATG
GACTCCGAGGCCCACATTGTCTCCATCGAGGCCCACGACGGCGTCGTCGACATCGT
TGCCAATGGTAACCTCTGGGCTGATGGCCTCCGCGTCTACAACGTCAGCAACATCC
GTGTGCGCATTGTTGCTGGCGCCGCCCCTGCTGCTGCTGCTGCTGCTGCTGCTGTTG
CTGCTCCGGCTGCCGCCCCTGCTCCGGTTGCTGCATCTGGCCCTGCCCAGACCATCA
CCCTCAAGCAGCTCAAGGCTGAGCTTCTTGACGTTGAGAAGCCTCTCTACATCTCCT
CCAGCAACGGCCAGGTCAAGAAGCACGCCGATGTGGCTGGTGGCCAGGCCACCAT
TGTGCAGGCTTGCAGCCTCAGTGACCTCGGTGATGAAGGCTTCATGAAGACCTACG
GTGTTGTGGCTCCTCTCTACACCGGTGCCATGGCCAAGGGTATTGCCTCTGCTGACC
TTGTGATTGCCACTGGTAAGCGCAAGATCCTCGGTTCCTTCGGTGCTGGCGGTCTCC
CCATGCACATTGTCCGTGCCGCTGTTGAGAAGATCCAGGCTGAGCTCCCGAACGGC
CCCTTCGCCGTCAACCTCATCCACTCCCCCTTCGATAGCAACCTTGAGAAGGGCAA
CGTTGACCTCTTCCTCGAGAAGGGCGTTACTGTCGTCGAGGCCTCCGCCTTCATGAC
CTTGACCCCGCAAGTCGTCCGCTACCGTGCTGCTGGTCTTTCCCGTAACGCTGATGG
CTCCATTAACATCAAGAACCGCATCATCGGTAAGGTCTCCCGTACCGAGCTCGCTGA
GATGTTCATCCGCCCTGCCCCGCAGAACCTCCTCGACAAGCTCATCCAGTCTGGTG
AGATTACCAAGGAGCAGGCTGAGCTTGCCAAGCTCGTCCCCGTCGCCGACGACATC
GCCGTCGAGGCCGACTCTGGTGGCCACACCGACAACCGCCCCATCCACGTCATCCT
CCCCCTTATCATCAACCTCCGCAACCGCCTCCACAAGGAGTGCGGCTACCCCGCTC
ACCTCCGCGTGCGCGTTGGAGCTGGTGGTGGTGTTGGATGCCCCCAGGCCGCTGCC
GCTGCTCTCGCTATGGGTGCTGCCTTCCTTGTTACCGGCACTGTCAACCAGGTCGCC
AAGCAGTCCGGCACCTGCGACAATGTCCGCAAGCAGCTCTGCATGGCCACCTACTC
TGACGTCTGCATGGCTCCCGCTGCTGACATGTTCGAGGAGGGCGTCAAGCTCCAGG
TCCTCAAGAAGGGAACCATGTTCCCGTCCAGGGCTAA

Table S4. Primers used in RT-qPCR for detect expression of the genes

Primers	Sequence(5′-3′)
FAS-F	TGAGGCGTTTGCCAACTACA
FAS-R	GCTCCTTGACAATGACCATG
OrfB-ER-F	GTTGAAGCCTCCGCCTTTATG
OrfB-ER-R	CCTGCTCTTGGGTGATCTCGC
OrfC-ER-F	CAGCCTGCTCCTTGGTAATCT
OrfC-ER-R	CATCAAGAACCGCATCATCG
DH-F	AGCCTCGTCGTAGCCCATCT
DH-R	TGCAGCCAGCTCCTCAAGAT
